# Clinical characteristics analysis of stroke patients with lower extremity deep venous thrombosis

**DOI:** 10.3389/fneur.2025.1635292

**Published:** 2026-01-09

**Authors:** Changbin Liu, Anming Hu, Sihao Liu, Zhaoxia Wang, Huixian Yu, Pei Dai, Huiqing Hou, Yumei Zhang, Dawei Zang

**Affiliations:** Department of Rehabilitation Medicine, Beijing Tian’tan Hospital, Capital Medical University, Beijing, China

**Keywords:** stroke, deep venous thrombosis of lower extremities, rehabilitation therapy, anticoagulation therapy, clinical characteristic

## Abstract

**Objective:**

To analyze the clinical characteristics of stroke patients with lower extremity deep venous thrombosis (DVT), and to provide a reference for the prevention and treatment of DVT in stroke patients.

**Methods:**

A retrospective analysis was performed on the clinical records of 5,980 patients diagnosed with stroke and admitted to Beijing Tian’tan Hospital, Capital Medical University, between January and December 2023. Based on the presence or absence of DVT, patients were categorized into two groups: a control group (without DVT, *n* = 5,862) and a DVT group (with DVT, *n* = 118). Propensity score matching was conducted at a 1:1 ratio, yielding 118 matched cases in each group. Comparative analyses were conducted to evaluate the clinical characteristics of the matched cohorts. Multivariate logistic regression analysis was employed to identify independent risk factors associated with the development of lower extremity DVT in stroke patients. Additionally, the functional status, treatment strategies, and clinical outcomes of patients with stroke complicated by lower extremity DVT were systematically assessed.

**Results:**

The incidence of lower extremity DVT was significantly higher in stroke patients aged ≥65 years, those with bed rest duration ≥3 days, a history of smoking, absence of anticoagulant therapy, muscle strength < grade 3, and without rehabilitation therapy (*p* < 0.05). Additionally, comorbid conditions such as diabetes mellitus, hypertension, hyperhomocysteinemia (Hcy), elevated D-dimer, hypertriglyceridemia, and increased low-density lipoprotein (LDL) cholesterol were also significantly associated with a higher incidence of lower extremity DVT (*p* < 0.05). Multivariate logistic regression analysis identified all of these variables as independent risk factors for the development of lower extremity DVT in stroke patients (*p* < 0.05).

**Conclusion:**

Vigilance for DVT is warranted in this high-risk population. Implementation of early, systematic therapeutic interventions (rehabilitation and appropriate anticoagulation therapy) following stroke is recommended to mitigate the incidence of lower extremity DVT.

## 1 Introduction

Stroke is a prevalent cardiovascular and cerebrovascular disorder that encompasses both hemorrhagic and ischemic subtypes. It frequently occurs as a complication of underlying conditions such as hypertension, diabetes mellitus, atrial fibrillation, and cerebral aneurysms (1). Stroke often results in neurological impairments, including motor dysfunction, sensory deficits, cognitive impairment, dysphagia, and aphasia. In the absence of timely medical intervention, it can lead to severe disability or death within a short time frame. Among these sequelae, motor dysfunction is the most common clinical manifestation and represents a primary contributor to long-term disability in stroke survivors (2, 3).

At present, with the maturity of the clinical rescue system and the development of early rehabilitation therapy technology, an increasing number of stroke patients are able to receive timely medical intervention. However, a substantial proportion of patients continue to experience limb hemiplegia following stroke onset (4). Hemiplegia often results in reduced muscle strength and tone in the affected limbs. When combined with prolonged bed rest, this leads to reduced mobility and insufficient physical activity, predisposing patients to lower extremity deep venous thrombosis (DVT) (5). The formation of DVT in the lower extremities impedes venous return, causing venous congestion, increased intravascular pressure, and subsequent tissue swelling and pain. If left untreated, chronic DVT may lead to complications such as skin pigmentation changes and venous ulcers. Critically, detachment of the thrombus can result in pulmonary embolism, a potentially life-threatening condition (6, 7). Early systematic therapy has a significant effect on the prevention and treatment of DVT, and early prevention and timely detection of lower extremity DVT are of great significance to stroke patients The purpose of the present study was to analyze the clinical characteristics of stroke patients with lower extremity DVT, and to provide reference data for the systematic and treatment of stroke patients with lower extremity DVT.

## 2 Materials and methods

### 2.1 Clinical data

A retrospective analysis was conducted on the clinical data of 5,980 stroke patients admitted to our hospital between January and December 2023. Patients were categorized into a control group [without deep vein thrombosis (DVT), *n* = 5,862] and a study group (with DVT, *n* = 118) based on the presence of lower extremity DVT. Propensity score matching was applied at a 1:1 ratio, the matching covariates were described in observation indicators section and the caliper was <0.05, resulting in 118 matched cases in each group for comparative analysis. Inclusion criteria for the study were: (1) diagnosis of stable stroke, including both hemorrhagic and ischemic subtypes, complicated by DVT; (2) normal consciousness and ability to cooperate with clinical assessments; and (3) absence of trauma, muscular diseases, or malignancies. Exclusion criteria were: (1) impaired consciousness that interfered with cooperation; (2) unstable clinical condition; (3) presence of musculoskeletal disorders or history of limb surgery; (4) neuromuscular diseases; (5) malignant tumors; and (6) coagulation dysfunction. The study protocol was reviewed and approved by the institutional medical ethics review committee (Ethics Approval Number: KY2020-069-01).

### 2.2 Methods

The diagnosis of lower extremity DVT is primarily based on the findings of lower extremity venous ultrasonography. We perform lower extremity venous ultrasound examinations on patients at the time of admission and discharge. If the ultrasound results indicate DVT at the time of admission, we perform a follow-up vascular ultrasound once a week. A diagnosis of DVT is established when the ultrasound reveals the presence of echogenic material within the venous lumen, indicating a solid thrombus, accompanied by interruption or absence of color Doppler blood flow signals at the site of the thrombus. Additional ultrasonographic features supporting the diagnosis include intraluminal filling defects and localized narrowing or collapse of the venous lumen (8, 9).

### 2.3 Observation indicators

The observation indicators in the present study included several key components. First, basic demographic and clinical information of stroke patients was collected, including age, sex, history of smoking and alcohol consumption, underlying comorbidities, laboratory test results, imaging findings, and stroke type. Second, functional impairments in stroke patients with lower extremity DVT were evaluated. Third, the clinical characteristics of patients in the DVT group and the control group were comparatively analyzed. Fourth, multivariate logistic regression analysis was performed to identify independent risk factors associated with the development of lower extremity DVT in stroke patients. Finally, the study assessed preventive measures, treatment strategies, and prognostic outcomes in patients with stroke complicated by lower extremity DVT. We also defined some variables based on the laboratory tests conducted by the hospital, high Hcy means the Hcy value > 15 μmol/L, high D-d means the D-d value > 1.50 μg/mL, high TG means the TG value > 1.70 mmol/L, high LDL means the LDL value > 3.10 mmol/L.

### 2.4 Statistical analysis

Statistical analysis was performed using GraphPad Prism 9.0 software. Measurement data were expressed as *x-* ± s, and the groups were compared through analysis using the independent sample t-test. Count data were expressed as cases (%), and the groups were compared through analysis using the χ^2^ test. Logistic analysis was used to analyze the risk factors and accompanying symptoms of DVT after stroke, and *p* < 0.05 was considered statistically significant.

## 3 Results

### 3.1 Analysis of functional disorders in patients with stroke complicated with lower extremity DVT

In total, 118 cases of lower extremity DVT were found in 5,980 stroke patients (1.97%), aged from 4 to 85 years, including 64 males (54.24%) and 54 females (45.76%). Among those with DVT, there were 89 cases of ischemic stroke (75.24%), 29 cases of hemorrhagic stroke (24.7%), 118 cases with motor disorders (100%), 28 cases with aphasia (23.73%), 28 cases with disorders (23.73%), 65 cases with dysarthria (55%), 65 cases with dysphagia (55%), and 26 cases with sensory disorders (22.03%).

### 3.2 Comparison of clinical characteristics between the two groups of patients

There were no statistically significant differences between the study and control groups in terms of gender, BMI, history of drinking, or stroke type (*p* > 0.05). However, the incidence of lower extremity DVT was significantly higher in patients aged ≥65 years, with bed rest time ≥3 days after admission, a history of smoking, no anticoagulation therapy, reduced muscle strength <3 grades, and absence of rehabilitation therapy. Additionally, patients with diabetes, hypertension, high Hcy, high D-dimer, high TG, and high LDL were more commonly observed in the study group compared to the control group (*p* < 0.05). Detailed data are shown in Table 1.

**Table 1:** Comparison of clinical characteristics between the two groups of patients.

Indicator	Study group	Control group	*χ* ^2^	*P*
Gender (male/female)	64/54	62/56	*0.122*	*0.793*
Age (≥65 years/<65 years)	76/42	36/82	4.133	0.034
BMI (kg/m^2^)	24.12 ± 4.35	23.83 ± 3.21	0.253	0.797
Drinking (yes/no)	61/57	55/63	0.113	0.802
Smoking (yes/no)	81/37	43/75	3.454	0.039
Stroke type (hemorrhagic/ischemic)	29/89	36/82	0.172	0.719
Bedtime (≥3 days/<3 days)	89/29	23/95	6.813	0.015
Anticoagulation (yes/no)	25/93	62/56	4.697	0.028
Muscle strength < grade 3 (yes/no)	42/76	18/100	4.148	0.032
Rehabilitation (yes/no)	16/102	89/29	7.957	0.006
Diabetes (yes/no)	71/47	53/65	2.648	0.042
Hypertension (yes/no)	106/12	81/37	2.148	0.047
High Hcy (yes/no)	79/39	41/77	3.441	0.039
High D-d (yes/no)	107/11	26/92	7.015	0.009
High TG (yes/no)	93/25	68/50	2.703	0.041
High LDL (yes/no)	95/23	72/46	2.542	0.043

### 3.3 Analysis of independent influencing factors of lower extremity DVT in stroke patients

The results of multivariate logistic regression analysis indicated that age ≥65 years, bed rest time ≥3 days after admission, history of smoking, absence of anticoagulation therapy, muscle strength < grade 3, lack of rehabilitation therapy, comorbid diabetes, comorbid hypertension, elevated Hcy, D-dimer, TG, and LDL levels were independent risk factors for the development of lower extremity DVT in stroke patients (*p* < 0.05). Detailed results are presented in Table 2.

**Table 2:** Analysis of independent influencing factors of lower extremity DVT in stroke patients.

Variable	β	Wald *χ*^2^	SE	OR	95%CI	*P*
Age	0.469	7.476	0.627	1.246	0.302 ~ 6.519	0.014
Bedtime	2.612	10.725	0.701	9.132	3.631 ~ 39.615	<0.001
Smoking	1.124	9.618	0.481	7.621	1.936 ~ 23.112	0.002
Anticoagulation	3.612	15.725	0.907	13.139	5.631 ~ 38.625	<0.001
Muscle strength	5.602	18.725	0.987	21.139	4.603 ~ 40.675	<0.001
Rehabilitation	4.512	16.725	0.883	16.179	3.643 ~ 36.517	<0.001
Diabetes	1.439	3.476	0.427	2.144	0.409 ~ 5.519	0.004
Hypertension	0.461	5.476	0.728	1.198	0.624 ~ 6.509	0.009
High Hcy	0.279	6.408	0.927	2.946	0.612 ~ 9.589	0.023
High D-d	6.003	21.822	0.786	26.038	3.393 ~ 57.603	<0.001
High TG	0.499	6.406	0.627	1.194	0.308 ~ 4.419	0.012
High LDL	0.316	7.817	0.522	2.323	0.297 ~ 5.613	0.019

The scoring of each factor was age (<65 years = 0, ≥65 years = 1); smoking (no = 0, yes = 1); hemiplegia time ≥3 d (no = 0, yes = 1); absence of anticoagulation (no = 0, yes = 1); muscle strength <3 grades (no = 0, yes = 1); rehabilitation (no = 0, yes = 1); diabetes (no = 0, yes = 1); hypertension (= 0, yes = 1); high Hcy (no = 0, yes = 1); high D-d (no = 0, = 1); high TG (no = 0, yes = 1); high LDL (no = 0, yes = 1).

## 4 Discussion

In the present study, 5,980 patients with stroke were included, of which 118 (1.97%) had lower extremity DVT. The occurrence of DVT in stroke patients not only increases healthcare costs but also significantly hinders the rehabilitation process. In severe cases, it may lead to pulmonary embolism and even death (6). The pathogenesis of DVT primarily involves three key mechanisms: endothelial injury, venous stasis, and a hypercoagulable state. Venous stasis contributes to local tissue hypoxia, which in turn promotes the accumulation of thrombin, cellular injury, and the release of vasoactive substances such as serotonin and histamine, thereby initiating thrombus formation. Moreover, surgical interventions or prolonged bed rest can further reduce blood flow velocity, facilitating thrombosis. Under normal physiological conditions, the endothelial lining of venous vessels provides a natural antithrombotic barrier. Endothelial cells are coated with heparin and secrete anticoagulant substances, including prostaglandins and other regulatory proteins, to inhibit thrombus formation. A hypercoagulable state can arise from both congenital and acquired factors. Congenital factors include deficiencies in natural anticoagulants (e.g., thrombin inhibitors), abnormal fibrin formation, and impaired fibrinolysis, while acquired factors involve surgery, pharmacological agents, and underlying conditions such as diabetes mellitus (10–13).

In the present study, all stroke patients exhibited motor dysfunction (100%), and the findings indicated that bed rest for more than 3 days was an independent risk factor for the development of lower extremity DVT. During the acute phase of stroke, patients are often in an unstable condition, and clinical management primarily focuses on secondary stroke prevention. This includes the control of underlying diseases, neuroprotective therapy, and enhancement of cerebral circulation. As a result, patients are frequently confined to bed and receive predominantly passive treatment, which contributes to prolonged immobility and venous stasis, key factors in the pathogenesis of DVT. The results show that the muscle strength of the affected lower limb was less than grade 3, which is an independent risk factor for DVT. Patients with muscle strength less than 3 are unable to perform movements against gravity and are limited to in-bed limb movements or simple isometric contractions. In contrast, those with muscle strength greater than grade 3 can actively lift their limbs off the bed and engage in broader, functional joint movements. As a result, the risk of lower extremity DVT is significantly lower in patients with greater muscle strength due to improved venous return facilitated by active mobility. Studies have shown that lack of rehabilitation therapy is an independent risk factor for DVT. Rehabilitation therapy, including physical therapy, use of thrombosis pumps, and in-bed exercises, plays a crucial role in enhancing limb muscle strength, improving joint range of motion, and promoting blood circulation, thereby effectively reducing the risk of lower extremity DVT. In the present study, the incidence of lower extremity DVT was found to be 1.97%, which is slightly lower than the rates reported in previous studies. A possible explanation for this discrepancy is that our hospital specializes in neurology, and the overall quality and effectiveness of stroke treatment and rehabilitation are relatively high. The concept of rehabilitation medicine is fully integrated into the clinical workflow of our hospital, with rehabilitation therapy routinely initiated once the patient’s condition stabilizes. Early bedside rehabilitation is implemented in a timely manner, contributing to improved functional recovery. Additionally, patients admitted to the rehabilitation department predominantly originate from the hospital’s own neurology and neurosurgery departments, ensuring seamless continuity between clinical management and rehabilitative care. This integrated approach enhances the overall quality of care and likely contributes to the observed reduction in the incidence of lower extremity DVT. Therefore, early and systematic rehabilitation therapy is of critical importance in the prevention of lower extremity DVT in stroke patients.

In the present study, D-dimer was found to be an independent risk factor for the occurrence of DVT in stroke patients, and multiple studies have also identified Dimer as a strong risk factor and diagnostic marker for DVT (10–13). D-dimer is a fibrin degradation product (FDP) generated during the breakdown of fibrin clots by the fibrinolytic system. It represents a specific marker of thrombus formation and subsequent fibrinolysis. Therefore, elevated D-dimer levels in the blood indicate active coagulation and fibrinolysis processes (14). Previous studies have found that D-dimer can increase the risk of DVT by 3.5 times (15). Clinically, elevated D-dimer levels are commonly used as an indicator of thrombosis, as they reflect ongoing activation of the coagulation and fibrinolytic systems. However, D-dimer is not specific to venous thrombosis and may also be elevated in a variety of other pathological and physiological conditions, including malignancies, systemic inflammation, pregnancy, liver disease, recent surgery, and trauma (16). As such, D-dimer cannot be used as the sole indicator for the diagnosis of DVT, and it should be combined with vascular ultrasound for diagnosis (17). The absence of anticoagulation therapy has been identified as an independent risk factor for the development of lower extremity DVT. Anticoagulation therapy, particularly with agents such as low molecular weight heparin, is widely regarded as the first-line treatment for both the prevention and management of lower extremity venous thrombosis in clinical practice. Low molecular weight heparin offers several pharmacological advantages, including a long half-life, high bioavailability, and a potent antithrombotic effect (18, 19). Studies have demonstrated that low molecular weight heparin calcium significantly enhances coagulation function in patients with lower extremity venous thrombosis and contributes to alleviation of disease severity (20). Stroke includes both ischemic and hemorrhagic subtypes. In patients recovering from ischemic stroke, low molecular weight heparin may be administered, depending on the clinical condition, to prevent the development of lower extremity DVT. However, the potential risk of bleeding must be carefully evaluated prior to initiation. In contrast, for patients with hemorrhagic stroke, particularly those with incomplete hematoma absorption, the preventive use of low molecular weight heparin is generally not recommended due to the heightened risk of secondary intracranial bleeding.

The findings of the present study indicate that advanced age is a significant risk factor for lower extremity DVT, with individuals aged 65 years and older generally classified as elderly. The incidence of stroke is higher in this population, and elderly patients often present with multiple chronic comorbidities, which further contribute to thrombus formation. With increasing age, the vascular endothelium undergoes structural deterioration, becoming damaged, aged, and roughened, creating a favorable environment for thrombogenesis. Existing literature supports that age over 65 is a major risk factor for the development of DVT following stroke, with the incidence rising progressively with advancing age (21). This finding is further supported by the results of the present study, which showed that, according to multivariate logistic regression analysis, the risk of developing lower extremity DVT in stroke patients increased by 3.08 times for each additional year of age beyond 65. Thus, it can be concluded that elderly stroke patients are at significantly higher risk of DVT, and the incidence of DVT increases progressively with advancing age (22).

In the present study, the independent factors of post-stroke DVT were also found to include smoking, comorbid diabetes, comorbid hypertension, high Hcy, high TG, and high LDL in the multifactorial logistic regression prediction scale. Previous studies have demonstrated that these factors contribute to alterations in vascular morphology and damage to endothelial cells, thereby promoting thrombus formation in the extremities. Notably, these same factors are also well-established risk factors for the onset of stroke itself (17, 19, 23–25). However, the present study has certain limitations, including a relatively limited sample size and its single-center design. Emphasize the need for multicenter validation and acknowledge the limitations of retrospective data, particularly regarding unmeasured confounding (e.g., mobility protocols, thromboembolism prophylaxis routines). Future research should aim to include larger patient cohorts and adopt a multi-center approach to enhance the generalizability and robustness of the findings.

## 5 Conclusion

In summary, identifying the influencing factors associated with the occurrence of lower extremity DVT in stroke patients enables healthcare providers to more accurately and efficiently assess individual patient risk and implement timely, targeted preventive measures. Interventions such as early and systematic rehabilitation therapy, minimizing prolonged bed rest, and appropriate anticoagulation therapy are crucial in reducing the incidence of DVT among stroke patients. These strategies are of significant clinical importance, as they contribute to shorter hospital stays, improved quality of life, and reduced mortality in this vulnerable population.

## Data Availability

The original contributions presented in the study are included in the article/supplementary material, further inquiries can be directed to the corresponding author.
